# Therapeutic Efficacy of Novel HDAC Inhibitors SPA3052 and SPA3074 against Intestinal Inflammation in a Murine Model of Colitis

**DOI:** 10.3390/ph15121515

**Published:** 2022-12-05

**Authors:** Ji-In Yoon, Hyewon Cho, Raok Jeon, Mi-Kyung Sung

**Affiliations:** 1 Department of Food and Nutrition, Sookmyung Women’s University, Seoul 04310, Republic of Korea; 2 College of Pharmacy, Sookmyung Women’s University, Seoul 04310, Republic of Korea

**Keywords:** colitis, HDAC, tight junction protein, occludin, SOCS1

## Abstract

Inflammatory bowel diseases (IBD) are digestive tract disorders that involve chronic inflammation with frequent recurrences. This study aimed to evaluate the efficacy of two novel histone deacetylase 8 (HDAC8) inhibitors, namely, SPA3052 and SPA3074, against dextran sulfate sodium (DSS)-induced experimental colitis. Male C57BL/6N mice were subjected to two cycles of 1.5% DSS followed by treatment with suberoylanilide hydroxamic acid (SAHA), SPA3052, or SPA3074 for 14 days. Our results showed that SPA3074 administration increased (>50%) the expression of occludin, a tight junction protein, which was significantly decreased (>100%) after DSS treatment. Moreover, SPA3074 upregulated suppressor of cytokine signaling 1 (SOCS1) protein expression, which is known to be a key suppressor of T-helper cell differentiation and pro-inflammatory cytokines expression. Furthermore, we observed a decrease in SOCS1-associated Akt phosphorylation and an increase in lower extracellular signal-regulated kinase 1 and 2 phosphorylation, which contributed to lower nuclear factor-kappa B activation. Th2 effector cytokines, especially interleukin-13, were also downregulated by SPA3074 treatment. This study suggests that HDAC8 might be a promising novel target for the development of IBD treatments and that the novel HDAC8 inhibitor SPA3074 is a new candidate for IBD therapeutics.

## 1. Introduction

Inflammatory bowel diseases (IBD) are chronic idiopathic relapsing disorders which cause inflammation of the gastrointestinal tract [1,2]. The two major forms of IBD are Crohn’s disease (CD) and ulcerative colitis (UC). The development of both UC and CD is known to be associated with various genetic and environmental factors [2]. Frequent exposure to certain environmental factors contributes to the development and progression of IBD in association with epigenome [3] and dysbiosis-induced disturbances in immune regulation [4]. Therefore, epigenetic changes due to gene–environment interactions have been suggested as important therapeutic targets for IBD. Many clinical trials which evaluated the efficacy of newly developed drugs regulating DNA methylation and histone deacetylation/acetylation have been conducted. However, most of the tested drugs caused serious side effects, such as diarrhea, fatigue, nausea, and anorexia [5,6].

Among others, epigenetic modifications of histones, particularly acetylation, have been suggested to be associated with the expression of genes which regulate inflammation [7]. Histone acetylation by histone acetyltransferases (HATs) and histone deacetylation by histone deacetylases (HDACs) are representative epigenetic mechanisms that modify chromatin through the hydrolysis of an acetyl moiety from the lysine residue [8], thereby influencing gene transcriptional activation [9]. In particular, HDACs have been shown to promote inflammatory responses and regulate innate and adaptive immune pathways [10]. Based on these findings, HDAC inhibitors (HDACis) have been considered as promising drug targets for the treatment of inflammatory diseases, such as rheumatoid arthritis, asthma, and cystic fibrosis lung disease [11,12,13,14]. HDACis also have been discovered and developed for the treatment of various cancers. Several studies have suggested that the mechanisms which underlie the effect of HDACis on IBD development include the control of cell proliferation and differentiation, protection of intestinal epithelial barrier, regulation of transcription factors, and control of the intestinal immune system [15].

Among HDACis, suberoylanilide hydroxamic acid (SAHA), also known as vorinostat, was first approved by the U.S. Food and Drug Administration to treat cutaneous T-cell lymphoma (CTCL) [16]. SAHA is also known to possess anti-inflammatory potential [17] and has been widely used as a positive control compound in the search for novel HDACis. In a previous study, inhibition of HDACs by SAHA was shown to attenuate inflammatory changes caused by dextran sulfate sodium (DSS)-induced colitis by suppressing the local secretion of pro-inflammatory cytokines and chemokines, as well as by inhibiting the mobilization and accumulation of inflammatory cells [18].

HDAC8, a class I HDAC, has been studied as an emerging therapeutic target in various diseases, including cancer, viral infectious diseases, and Cornelia de Lange syndrome [19]. Recently, specific inhibition of HDAC8 has been proven to regulate signaling pathways involved in inflammatory responses [19]. HDAC8 inhibition was shown to alter Janus kinase 2/signal transducer and activator of transcription (JAK2/STAT) signaling [20] and upregulate suppressor of cytokine signaling 1/3 (SOCS1/3) gene expression [21]. SOCS1 and 3 protein upregulation were also found to control T-cell development and ameliorate intestinal inflammation [22,23]. Moreover, specific inhibition of HDAC8 was shown to reduce the secretion of pro-inflammatory cytokines, namely, interleukin (IL)-6, tumor necrosis factor α, and IL-1β, and restore that of the anti-inflammatory cytokine IL-10 [24,25,26].

Previously, we discovered two novel organosulfur compounds, namely, SPA3052 and SPA3074, which act as selective HDAC8 inhibitors. In this study, we hypothesized that the novel HDAC8 inhibitors, SPA3052 and SPA3074, might be possible therapeutics for IBD and evaluated their efficacy and underlying mechanisms of action using a DSS-induced colitis murine model.

## 2. Results

### 2.1. Body Weight, Colon Shortening, and DAI Score

**Figure 1:**
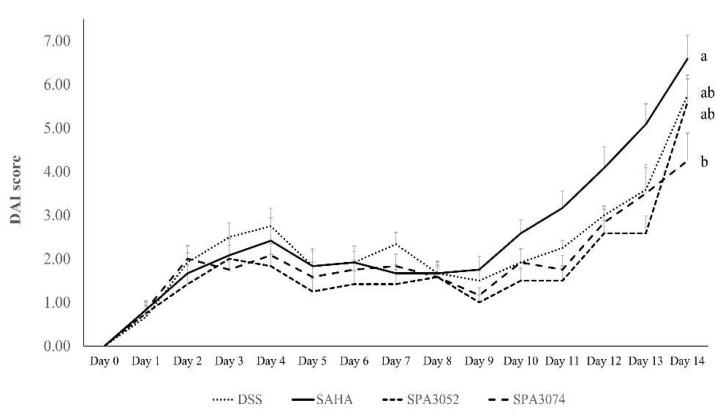
DAI score during 14 days of DSS administration in DSS. DAI was counted with the assessment of percentage of weight loss, stool consistency and fecal bleeding; Abbreviations: DAI = disease activity index; CON = control; DSS = dextran sulfate sodium; SAHA = suberoylanilide hydroxamic acid; All data represented mean ± S.E.M; *n* = 7–12 mice per group; ^abc^ Means with different superscripts are significantly different based on the Duncan’s multiple range tests (*p* < 0.05).

The colitis activity in groups treated with DSS was assessed using the disease activity index (DAI) score (Figure 1). On the last day of the experiment, DSS administration was found to significantly increase the DAI score in all DSS-treated groups when compared to day 1 (*p* < 0.05). There was no significant difference in DAI scores between groups. However, the SPA3074 group tended to have a lower DAI score (4.25 ± 0.62) compared to the other three groups (DSS: 5.75 ± 0.46; SAHA: 6.58 ± 0.54; SPA3052: 5.58 ± 0.54). DSS administration also induced significant weight loss and colon shortening (Table 1), although no significant improvement was observed between experimental groups.

### 2.2. Histological Score and Expression of the Tight Junction Protein

**Figure 2:**
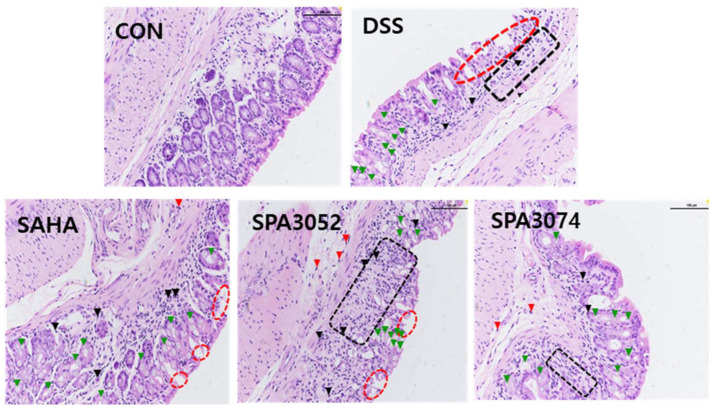
Representative images of H&E staining of colon sections. Black arrowheads and red arrowheads indicate leukocytes in mucosa and leukocytes in submucosa, respectively. Red dashed line area indicates loss of surface epithelium black dashed line area indicates mucosa devoid of crypts. Green arrowheads indicate goblet cells. Abbreviations: CON = control; DSS = dextran sulfate sodium; SAHA = suberoylanilide hydroxamic acid.

**Figure 3:**
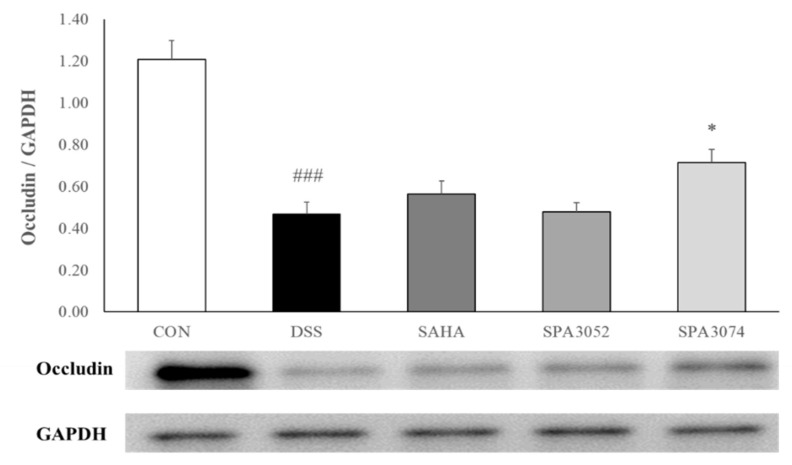
Protein expression of occludin was measured by Western blot; Abbreviations: CON = control; DSS = dextran sulfate sodium; SAHA = suberoylanilide hydroxamic acid; GAPDH= glyceraldehyde-3-Phosphate dehydrogenase; All data represented mean ± SEM.; *n* = 7–12 mice per group; ### *p* < 0.001 compared to the CON group; * *p* < 0.05 compared to the DSS group.

The colon H&E staining results (Figure 2) showed that DSS treatment increased inflammatory cell infiltration, as well as epithelial and mucosal architecture changes. However, administration of SPA3074 suppressed the erosions of epithelial cells and the loss of goblet cells, which resulted in a lower histological score (Table 2). The presence of the tight junction protein occludin has a crucial role in maintaining the integrity of the intestinal barrier [27]. As shown in Figure 3, DSS-induced colitis downregulated occludin expression, while the administration of SPA3074 showed significantly higher occludin expression levels, indicating that administration of SPA3074 suppressed DSS-induced occludin loss.

### 2.3. SOCS1 and SOCS3 Protein Expression

**Figure 4:**
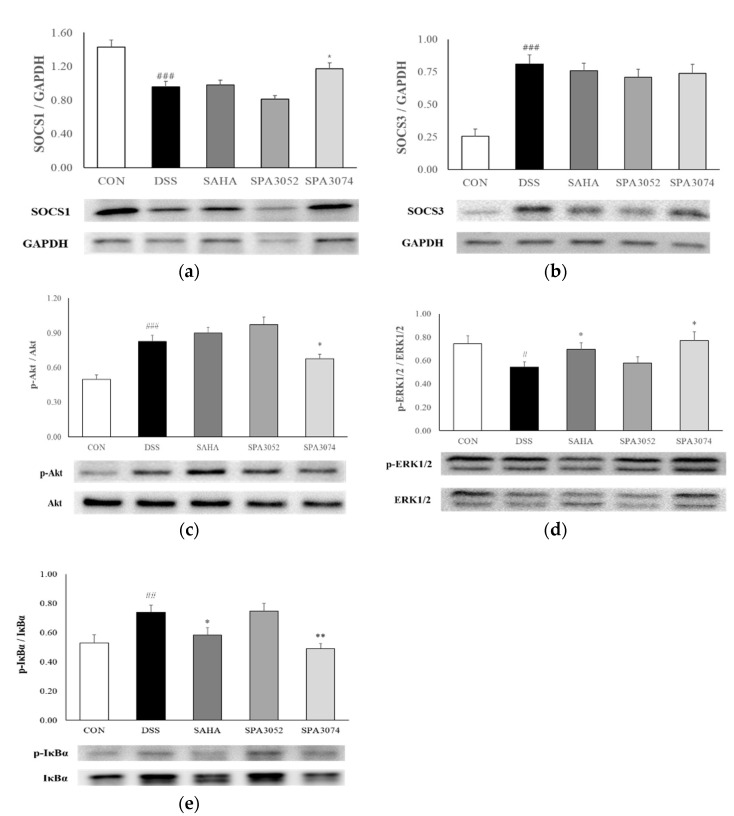
Protein expression of (**a**) SOCS1, (**b**) SOCS3, (**c**) phosphorylated-Akt and Akt, (**d**) phosphorylated-ERK1/2 and ERK1/2, (**e**) phosphorylated-IκBα and IκBα in colon tissue were measured by Western blot; Abbreviations: CON = control; DSS = dextran sulfate sodium; SAHA = suberoylanilide hydroxamic acid; SOCS = suppressor of cytokine signaling; ERK= extracellular signal-regulated kinase; GAPDH = glyceraldehyde-3-Phosphate dehydrogenase; All data represented mean ± S.E.M.; *n* = 7–12 mice per group; # *p* < 0.05, ## *p* < 0.01, ### *p* < 0.001 compared to the CON group; * *p* < 0.05, ** *p* < 0.01 compared to the DSS group.

To investigate the molecular mechanisms underlying the effects of SPA3074, the expression of the SOCS1 and SOCS3 proteins in colon tissues was determined (Figure 4a,b). The SOCS proteins are known to have the ability to suppress inflammation via negative regulation of transcription factors and cytokine signaling [28]. SOCS1 expression was found to be significantly decreased by DSS administration while SPA3074 treatment induced a significant increase in SOCS1 expression. However, DSS administration significantly upregulated the expression of SOCS3. No significant difference in expression level was observed between the treatment groups.

### 2.4. Protein Expression of Inflammation-Associated Mediators

The expression of the Akt (Protein kinase B) and extracellular signal-regulated kinase 1 and 2 (ERK1/2) proteins was determined in the colon tissue. Inhibition of the PI3K/Akt signaling pathway is known as a feasible therapeutic target for the treatment of chronic intestinal inflammatory disease [29]. The ratio between phosphorylated Akt and Akt in the DSS group was significantly increased when compared to that of the control group. However, SPA3074 administration significantly reduced the ratio (Figure 4c). ERK1/2 activity is known to play a protective role in response to proinflammatory cytokines and chemokine expression [30,31]. Mice treated with SAHA and SPA3074 showed a significantly higher p-ERK1/2 to ERK1/2 ratio when compared to the DSS group (Figure 4d). These results suggest that the test compounds, especially SPA3074, regulate important signaling molecules involved in inflammatory responses.

### 2.5. Protein Expression of Phophorylated Iκbα and IκBα

Phosphorylation or degradation of nuclear factor of kappa light polypeptide gene enhancer in B-cells inhibitor, alpha (IκBα) is a key step in the regulation of the nuclear factor-κB (NF-kB) pathway which results in the activation of inflammatory gene expression [32]. We analyzed the protein expression of p-IκBα and IκBα, as shown in Figure 4e, and found that the ratio between p-IκBα and IκBα was significantly increased by DSS administration, while SPA3074 and SAHA administration inhibited the phosphorylation of IκBα.

### 2.6. Relative mRNA Expression of Pro-Inflammatory Cytokines

**Figure 5:**
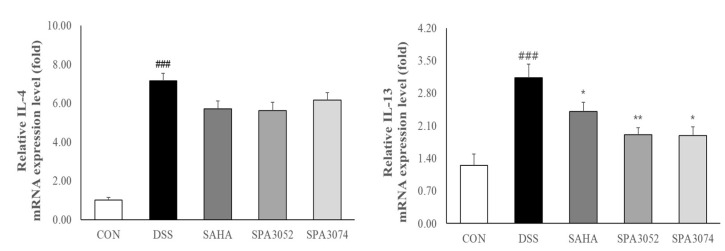
Relative mRNA expressions of IL-4 and IL-13 in colon tissue were measured by real-time PCR analysis; Abbreviations: CON = control; DSS = dextran sulfate sodium; SAHA = suberoylanilide hydroxamic acid; IL = interleukin; GAPDH = glyceraldehyde-3-Phosphate dehydrogenase; All data represented mean ± SEM.; *n* = 7–12 mice per group; ### *p* < 0.001 compared to the CON group; * *p* < 0.05, ** *p* < 0.01 compared to the DSS group.

DSS-induced colitis has been shown to induce T-helper cell 2 (Th2) immune responses in the gastrointestinal tract, with IL-4 and IL-13 being identified as important effector cytokines [33,34]. IL-4 and IL-13 expression was found to be significantly increased in the DSS group, while SAHA, SPA3052 or SPA3074 administration downregulated IL-13 expression (Figure 5). Moreover, all test compounds showed a tendency to suppress IL-4 mRNA expression, although statistically significant differences were not found.

## 3. Discussion

In spite of its unknown pathogenesis, various therapies have been suggested to manage IBD, mainly by blocking pro-inflammatory signaling and strengthening the epithelial barrier [35,36]. However, due to the unavoidable side effects of the currently used therapeutic drugs, continuous research efforts are greatly needed. As HDACis control gene expression by regulating the affinity between histones and DNA, many have been considered as drug candidates for the management of diseases associated with chronic inflammation. Recently, selective inhibition of HDAC8 has been suggested as a novel target, as it showed substantial anti-inflammatory potential. In this study, we determined the efficacy of two HDAC8 inhibitor candidates, namely, SPA3052 and SPA3074, against intestinal inflammation in a murine model of colitis. We found that SPA3074 administration upregulated SOCS1 expression compared to the DSS group in the colon tissue. The up-regulated SOCS1 may have reduced inflammatory damage to the colon epithelium possibly by suppressing Akt phosphorylation and promoting ERK1/2 phosphorylation, resulting in the inhibition of IκB activation in SPA3074 group. Moreover, SPA3074 group showed downregulated Th2 effector cytokine levels, especially IL-13 compared to the DSS group, thereby ameliorated inflammation.

IBD is considered to originate from an impaired mucosal barrier, which causes bacterial invasion because of increased intestinal permeability [37]. Subsequently, specific cytokines secreted from antigen-presenting cells stimulate the adaptive immunity, leading to T cell differentiation [38]. T cells differentiate into Th1, Th2, Th17, and Treg cells, and the balance between them controls the secretion of cytokines. The collapse of this balance and the dysregulated secretion of cytokines are known as representative IBD phenomena. In previous studies, selective HDAC6 inhibition was found to suppress the secretion of key inflammatory cytokines and chemokines in DSS-induced colitis [39]. Moreover, ITF2347 [40], SAHA, and valproic acid [41], which are pan-HDAC inhibitors, were shown to downregulate interferon (IFN)-γ and IL-6 levels, and upregulate the secretion of the anti-inflammatory cytokine IL-10. Among others, IL-4 and IL-13 are key effector cytokines in human UC and DSS-induced murine colitis. Notably, IL-13 was shown to induce epithelial apoptosis and loss of tight junction protein in patients with UC [42]. In this study, SPA3052 and SPA3074 administration significantly suppressed IL-13 mRNA expression, suggesting that HDAC8 inhibition may relieve intestinal inflammation by suppressing cytokine production.

To date, relatively few studies describing the effect of selective HDAC8 inhibition on IBD development or progression have been conducted, even though HDAC8 downregulates SOCS1, a key anti-inflammatory molecule. In this study, mice treated with SPA3074 showed an increase in colon SOCS1 expression compared to mice in DSS group, which might affect downstream signaling involving Akt and ERK1/2. In a previous study, reduced SOCS1 expression was observed in intestinal myofibroblasts derived from patients with UC [43]. Moreover, SOCS1-deficient mice exhibited dysregulated IL-4 and IFN-γ secretion in a rodent colitis model [22,44]. As SOCS1 plays a role in regulating T cell differentiation and cytokine expression [45], up-regulation of SOCS1 expression by inhibiting HDAC8 could be a promising strategy for the treatment of IBD. Unlike SOCS1, SOCS3 expression was increased by DSS administration. SOCS1 and SOCS3 were shown to be induced by IL-6 in inflamed mucosa and negatively regulate STAT/JAK activation, which is associated with the progression or development of intestinal inflammation [23]. However, the expression levels of SOCS1 and SOCS3 in experimental inflammatory disease models are controversial [22,46,47,48]. Considering the fact that SOCS3 was negatively regulated by the complex of SH2 domain-containing protein-tyrosine phosphatases 2 and tyrosine 759 in gp130, which does not affect SOCS1, SOCS3 is assumed to act as a feedback regulator for inflammation [49].

Due to the particular structural features of HDAC8, serine 39 phosphorylation by protein kinase A (PKA) inhibits the deacetylating activity of HDAC8 [50,51]. PKA triggers the phosphorylation of cAMP-response element binding protein (CREB) [52], which promotes FOXP3 expression and maintains the Treg phenotype [53], therefore playing a crucial role in the production of IL-10 [54]. Moreover, activation of PKA leads to mucin secretion in the colonic epithelium [55] that contributes to improving the protective function of the mucosal barrier [56]. In a previous study, PKA was shown to play a role in the translocation of ERK1/2, whose activation leads to the phosphorylation of CREB [57]. Activation of ERK1/2 promotes the production of IL-10, which induces an anti-inflammatory immune response and suppresses the expression of NF-kB-dependent pro-inflammatory genes [58,59]. The results of our study also showed that SAHA and SPA3074 treated animals exhibited significantly increased ERK1/2 activation compared to the DSS group animals, indicating that PKA may have played a role in ameliorating inflammation, which might be specifically medicated by HDAC8 inhibition.

In this study, SPA3074 and SPA3052 were tested in vivo to determine their efficacy in a mouse model of IBD. Of these two, only SPA3074 showed significant efficacy. This result is consistent with the result of the biochemical assay showing that SPA3074 possessed a more potent inhibitory activity against HDAC, with a lower IC50 value than SPA3052 (Supplementary Table S1). Moreover, in contrary to the expectation, the SAHA positive control group exhibited weight loss and a high DAI score at the end of the experiment. Indeed, diarrhea and weight loss are widely reported as major side effects of SAHA in cancer clinical trials and animal studies [27,60]. In fact, many studies used acute colitis model with relatively high doses of DSS administration [18,41] possibly escaping side effects of SAHA. In this study, however, low dose, multiple cycles of DSS were applied to mimic chronic inflammation which may have responded differently to SAHA. A limitation of this study is that the use of male mice only, mostly because estrogen is known to protect intestinal integrity in animal models of chemically-induced colitis.

In summary, this study shows that the novel HDAC8 inhibitor SPA3074 improved the histological score and maintained the tight junction protein levels, thereby strengthening the epithelial barrier in a murine model of colitis. Moreover, we found that an increase in SOCS1 protein levels and ERK1/2 phosphorylation along with spontaneous suppression of Akt and IκBα activation by SPA3074 might be the underlying molecular mechanisms of this effect.

## 4. Materials and Methods

### 4.1. Preparation of Chemical Compounds

**Figure 6:**
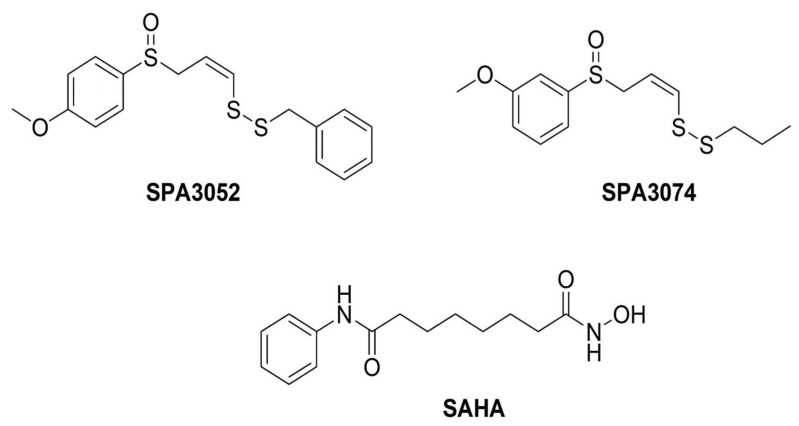
Chemical structure of the SPA3052 and SPA3074. SPA3052: (Z)-1-phenyl-7-(4-methoxyphenyl)-2,3,7-trithiahepta-4-ene-7-oxide; SPA3074: (Z)-9-(3-Methoxylphenyl)-4,5,9-trithianona-6-ene- 9-oxide; SAHA: suberoylanilide hydroxamic acid.

SPA3052 and SPA3074 were synthesized using the method reported by Hunter et al. [61]. Briefly, the thiol was propargylated and the radical reaction between the resulting propargylic thioether and thioacetic acid produced vinyl thioacetate. Deprotection and subsequent sulfenylation of the thioacetate resulted in vinyl disulfide, which was oxidized to generate the target compounds, SPA3052 and SPA3074. We selected these two compounds among several candidate compounds based on their superior inhibitory activities against human HDAC isoforms. SPA3052 and SPA3074 showed 85.9% and 88.2% inhibition towards HDAC8 activity at 20 μM concentration. The chemical structures and full names of SPA3052, SPA3074, and SAHA are shown in Figure 6. The pan-HDAC inhibitor SAHA was purchased from TCI Co. Ltd. (Tokyo Chemical Industry Co., Tokyo, Japan).

### 4.2. Animal Models of DSS-Induced Colitis

**Figure 7:**
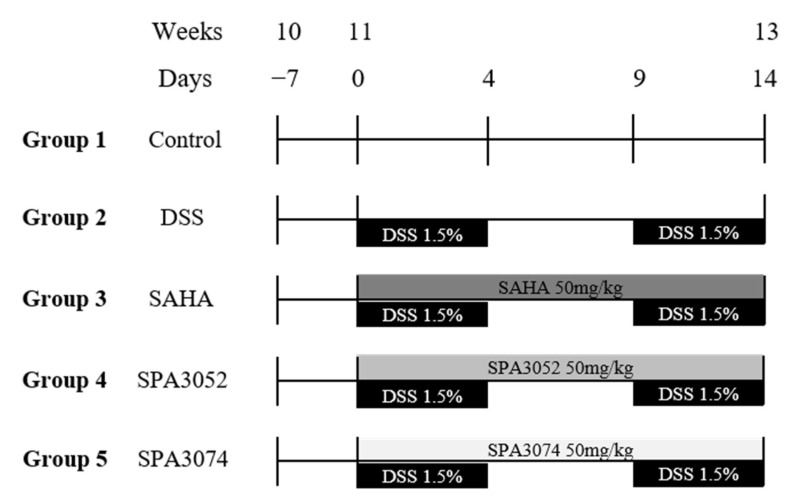
Experimental design of the experiments with the administration of SAHA, SPA3052, and SPA3074. Ten weeks old male C57BL/6N mice were used for the experiments. Two cycles of 1.5% DSS administration in performed for colitis induction. Supply of experimental compounds is maintained in the concentration of 50 mg/kg for whole experimental days; *n* = 7–12 mice per group.

Ten-week old male C57BL/6N mice purchased from Charles River Laboratories (Kanagawa, Japan) were housed in plastic cages (two to three mice per cage) under controlled temperature (21 ± 2 °C), humidity (50 ± 10%), and light (12 h light/dark cycle) conditions. After one week of adaptation, the animals were randomly divided into five groups as follows: Group 1 was the control group, treated with a vehicle (CON); Group 2 was the DSS control group, treated with 1.5% DSS in their drinking water (DSS) and vehicle; Group 3 was the SAHA group, treated with 1.5% DSS and 50 mg/kg/d SAHA; Group 4 was the SPA3052 group, treated with 1.5% DSS and 50 mg/kg/d SPA3052; Group 5 was the SPA3074 group, treated with 1.5% DSS and 50 mg/kg/d SPA3074. The concentrations of all compounds were decided according to previous in vivo studies using SAHA [41,62]. SAHA, SPA3052, and SPA3074 were dissolved in corn oil (Sajo Haepyo, Seoul, Republic of Korea) and administered by oral gavage, similar to other studies on HDACis (Figure 7) [63,64,65]. The DSS powder (MP Biomedicals, Solon, OH, USA) was dissolved in autoclaved tap water and replaced every two days. Animals were maintained for a further 15 days based on the AIN-93G diet (Research Diets, Inc, New Brunswick, NJ, USA) while they were subjected to 2 cycles (5 days and 4.5 days/cycle, respectively) of DSS treatment with 5 days between the two cycles. All of the procedures were approved by the Institutional Animal Care and Use Committee of Sookmyung Women’s University (SMWU-IACUC-1803-002) and followed the Korean Institutional Animal Care and Use Committee (IACUC) guidelines (Korea Food & Drug Administration, Chungju, Republic of Korea).

### 4.3. Disease Activity Index and Histological Observation

The disease activity index (DAI) was calculated by scoring weight loss and stool consistency and bleeding [66]. For weight loss, the score 0 was given to mice with no loss in body weight; score 1 for 1–5% loss in body weight; score 2 for 5–10% loss in body weight; score 3 for 10–20% loss in body weight; and score 4 for ≥ 20% loss in body weight. For stool consistency, the score 0 was given for normal, dry, and solid stool; score 2 for mild soft stool; score 3 for very soft stool; and score 4 for watery and diarrheic stool. For fecal bleeding, the score was 0 given for stool with no blood; score 1 for brown stool; score 2 for blooded stool; and score 3 for visible bleeding. Disease activity was measured daily during the experimental period. After the mice were euthanized, the rectums of five mice from each group were quickly removed and fixed in 10% formalin solution. The fixed colon segments were embedded in paraffin wax, cut, and then stained with hematoxylin and eosin (H&E) for histological analysis. The slides were observed using the Light microscope Olympus Provis AX70 (Olympus Optical Co., Tokyo, Japan) and the level of inflammatory cell infiltration, epithelial change, and mucosal architecture completion of the evaluated tissues were scored [67].

### 4.4. Real-Time PCR Analysis

Total RNA from the mouse rectum tissue was extracted using the TRIzol reagent (Invitrogen, Carlsbad, CA, USA), according to the manufacturer’s instructions. After spectrophotometric determination of RNA concentration and quality, the total RNA was converted to cDNA using a high-capacity cDNA Reverse Transcription Kit (PCR Biosystems, London, UK). Real-time PCR was performed in a volume of 20 μL using SYBR Green PCR Master Mix (PCR Biosystems, London, UK) and relevant primers. The reaction was performed using the following thermal cycling conditions: initial activation at 95 °C for 10 m, followed by 40 cycles of 15 s at 95 °C, 1 min at 60 °C, and 30 s at 95 °C, and then 15 s at 60 °C, using the ABI 7500 Fast instrument (Applied Biosystems, Foster City, CA, USA). Primers were designed using a nucleotide sequence and were synthesized by Bioneer (Bioneer, Daejeon, Republic of Korea). The following primers were used: IL-4 forward 5′-CCAGACTGCCTGCTTTTCAC-3′ and reverse 5′-CCATGGCTTGGGTACAGGTC-3′; IL-13, forward 5′-GGCACGAGTTTAAATGAGTCTGT-3′ and reverse 5′-CCAGAGCCCACTGCTTCAAT-3′. The relative quantification was performed using the Delta–Delta method [68]. The expression of the glyceraldehyde-3-phosphate dehydrogenase (GAPDH) housekeeping gene was used to normalize the results.

### 4.5. Protein Extraction and Western Blot Analysis

Colon tissue samples were homogenized for 20 min on ice using the protein extraction solution PRO-PREP^™^ (iNtRON Biotechnology, Gyeonggi, Republic of Korea) and then centrifuged (16,600× *g*, 10 min, 4 °C). The protein concentration was determined against a standardized control using a Bio-Rad Protein Assay kit (Bio-Rad Laboratories Inc., Hercules, CA, USA). A total of 40 µg of protein from each sample was separated by 4~20% or 4~12% sodium dodecyl sulfate-polyacrylamide gel electrophoresis and transferred to PVDF membranes (GE Healthcare, Mickleton, NJ, USA). The membranes were blocked with 4% skim milk (BD Biosciences, Franklin, NJ, USA) and incubated with specific antibodies against occludin (dilution 1:250; Abcam, Cambridge, MA, USA), SOCS1 (dilution 1:1000; Cell signaling, Boston, MA, USA), p-Akt (dilution 1:500; Cell signaling, Boston, MA, USA), Akt (dilution 1:1000; Cell signaling, Boston, MA, USA), p-IκBα (dilution 1:1000; Cell signaling, Boston, MA, USA), IκBα (dilution 1:1000; Cell signaling, Boston, MA, USA), ERK1/2 (dilution 1:1000; Cell signaling, Boston, MA, USA), and GAPDH (dilution 1:2500; Abcam, Cambridge, MA, USA) for a specific time, according to the manufacturer’s instructions. Anti-rabbit IgG or anti-mouse IgG antibodies conjugated with peroxidase (Sigma, St. Louis, MO, USA) were used as secondary antibodies. The membranes were washed with phosphate-buffered saline (PBS)/Tween-20 (PBST), containing 0.1% Tween-20 (Sigma, St. Louis, MO, USA). The reactive protein bands were visualized using an enhanced chemiluminescence (ECL) system (GE Healthcare, Mickleton, NJ, USA) and detected by LAS 3000 (Fujifilm, Tokyo, Japan). The expression level was determined by measuring the corresponding band intensities using the ImageJ software (National Institute of Health, Bethesda, MD, USA). GAPDH, a housekeeping gene, was used to normalize the immunoblots.

### 4.6. Statistical Analysis

Statistical analysis was performed using the SAS 9.4 package (SAS Institute Inc., Cary, NC, USA). The data are presented as mean values ± standard error of the mean (SEM). One-way analysis of variance followed by Duncan’s multiple range test were used to analyze the difference between the 5 experimental groups. Student’s *t*-test was used to analyze the significance between two groups when there is a tendency of difference without a statistical significance by Duncan’s multiple range test. Differences were considered statistically significant at *p* < 0.05.

## 5. Conclusion

This study suggests that HDAC8 is a promising new target for IBD treatment and the novel HAD C8 inhibitor SPA3074 a new candidate for IBD therapeutics.

## Figures and Tables

**Table 1:** Final body weight and colon length.

Group	Final Body Weight	Colon Length
CON	25.71 ± 0.88 ^a^	6.47 ± 0.15 ^a^
DSS	23.97 ± 0.33 ^b^	5.43 ± 0.19 ^b^
SAHA	22.76 ± 0.31 ^c^	5.28 ± 0.15 ^b^
SPA3052	23.96 ± 0.34 ^b^	5.42 ± 0.18 ^b^
SPA3074	24.15 ± 0.21 ^b^	5.46 ± 0.12 ^b^
*p* value	0.0005	0.0002

Abbreviations: CON = control; DSS = dextran sulfate sodium; SAHA = suberoylanilide hydroxamic acid; BW = body weight; All data represented mean ± SEM.; *n* = 7–12 mice per group; ^abc^ Means with different superscripts are significantly different based on the Duncan’s multiple range tests (*p* < 0.05).

**Table 2:** Histological scores.

Group	Inflammatory Cell Infiltrate	Epithelial Changes	Mucosal Architecture	Total Score
CON	0.67 ± 0.667	0.00 ± 0.000	0.00 ± 0.000	0.67 ± 0.667
DSS	3.60 ± 0.400 ^##^	6.60 ± 0.812 ^##^	3.40 ± 0.872 ^#^	13.60 ± 1.077 ^###^
SAHA	3.20 ± 0.374	4.00 ± 1.095	2.60 ± 1.077	9.80 ± 2.267
SPA3052	3.20 ± 0.200	5.00 ± 0.894	2.80 ± 1.158	11.00 ± 1.924
SPA3074	3.00 ± 0.000	4.00 ± 0.632 ^*^	3.40 ± 0.872	10.40 ± 0.748 ^*^

Abbreviations: CON = control; DSS = dextran sulfate sodium; SAHA = suberoylanilide hydroxamic acid; BW = body weight; All data represented mean ± SEM; *n* = 7–12 mice per group; # *p* < 0.05, ## *p* < 0.01, ### *p* < 0.001 in comparisons to the CON group; * *p* < 0.05 in comparisons to DSS group.

## Data Availability

Data is contained within the article and Supplementary Materials.

## Supplementary Materials

The following are available online at https://www.mdpi.com/article/10.3390/ph15121515/s1, Table S1: HDAC Enzyme inhibitory activity.
