# Supplementary material for: Therapeutic Efficacy of Novel HDAC Inhibitors SPA3052 and SPA3074 against Intestinal Inflammation in a Murine Model of Colitis
Source: Pharmaceuticals (Basel). 2022 Dec 5;15(12):1515. doi: 10.3390/ph15121515 (PMC9785328; doi:10.3390/ph15121515)
Supplement: Supplementary file 1 [file pharmaceuticals-15-01515-s001.zip › pharmaceuticals-2023133-supplementary.pdf]

Supplementary Table S1. HDAC Enzyme inhibitory activity

SPA	IC_{50} (μM)[a]	Human HDAC isoforms (% inhibition)[b]									
		1	2	3	4	5	6	7	8	9	11
3074	0.89	23.3	11.6	22.3	7.1	31.8	31.9	24.8	88.2	27.3	34.7
3052	1.34	42.5	10.9	22.6	16.1	27.7	51.4	21.1	85.9	28.1	24.0

[a]IC_{50} values are the mean if at least there are two experiments

[b]%inhibition at 20 μM
